# Chelidonine Induces Caspase-Dependent and Caspase-Independent Cell Death through G_2/M_ Arrest in the T98G Human Glioblastoma Cell Line

**DOI:** 10.1155/2019/6318179

**Published:** 2019-05-26

**Authors:** Yeon-Kyeong Lee, Ki Won Lee, Minju Kim, Yerin Lee, Jiyun Yoo, Cheol Hwangbo, Ki Hun Park, Kwang Dong Kim

**Affiliations:** ^1^Division of Applied Life Science (BK21Plus), Gyeongsang National University, Jinju 660-701, Republic of Korea; ^2^Division of Life Science, Gyeongsang National University, Jinju 660-701, Republic of Korea; ^3^PMBBRC, Gyeongsang National University, Jinju 660-701, Republic of Korea

## Abstract

*Chelidonium majus L*. (family Papaveraceae), commonly known as greater celandine or tetterwort, has been reported to have antibacterial and anticancer effects and chelidonine is known as a functional metabolite extracted from C.

## 1. Introduction

Glioblastoma is the most aggressive primary brain tumor and is classified as a grade IV astrocytoma in the central nervous system [1]. Although remarkable advances have been made in surgical techniques, radiotherapy, and chemotherapy, the treatment of glioblastoma remains extremely difficult and can extend the lives of patients by only a few months [2]. According to previous reports, the survival rate of patients is only 3% - 5%, and death usually follows after a mean period of 12-15 months [3]. The highly invasive nature of glioma is the result of the ability of the cancer cells to extensively proliferate and infiltrate between normal brain cells, which makes them elusive targets for effective surgical management. In addition, several mechanisms responsible for programmed cell death induction do not function, and molecular chaperones promoting cell survival are overexpressed in glioblastoma cells [4]. Therefore, the study of the signaling pathway inducing programmed cell death may be useful to identify the means of control that could provide novel cancer therapies for glioblastoma. Furthermore, alongside technological advances, scientific approaches based on traditional Chinese medical knowledge have provided a new direction for research into the treatment of cancers.

*Chelidonium majus L.* (family Papaveraceae) is widely used in traditional Chinese medicine.* C. majus* contains isoquinoline alkaloids (such as sanquinarine, chelidonine, chelerythrine, berberine, protopine, and coptisine), flavonoids, and phenolic acids as major compounds [5]. Crude extracts of this plant and purified compounds have been shown to have a wide range of biological activities [6], including anti-inflammatory [7], antimicrobial [8], immunomodulatory [9], anticancer [10], choleretic [11], hepatoprotective [12], and analgesic activities. Chelidonine is a major alkaloid compound of* C. majus* extracts, which exhibits anticancer and antiviral properties [5].

In this study, we investigated the mechanism of chelidonine-mediated cytotoxicity in the T98G human glioblastoma cell and conclude that chelidonine could be developed as an anticancer drug for use against aggressive glioblastoma.

## 2. Materials and Methods

### 2.1. Cell Culture

The human glioblastoma cell line, T98G, the breast cancer cell lines, MCF7 and MDA-MB-231, human embryonic kidney cell line, HEK293, human endothelial cell line, HUVEC, and human skin fibroblast cell line, CCD-25Sk, were purchased from the American Type Culture Collection (ATCC) (VA, USA). The lung cancer line A549 was purchased from the Korean Cell Line Bank (Seoul, Korea). Cells were grown as a monolayer culture in Dulbecco's Modified Eagle's Medium (Sigma-Aldrich, MO, USA) or RPMI 1640 (Sigma-Aldrich) supplemented with 10% (v/v) fetal bovine serum (Sigma-Aldrich) and 1% (v/v) penicillin/streptomycin (Lonza, Basel, Switzerland) solution at 37°C in an incubator containing 5% CO2.

### 2.2. Reagents and Antibodies

Chelidonine purchased from Sigma-Aldrich was used in this study. To inhibit apoptosis, the pan-caspase inhibitor Z-VAD-FMK (Enzo Life Sciences, Farmingdale, NY, USA) was used and dimethyl sulfoxide (DMSO) (Sigma-Aldrich) was used as a vehicle. For western blot analysis, the following antibodies were used: anticaspase-3, caspase-9, poly (ADP-ribose) polymerase (PARP), Mcl-1, BAK, p-CDK1(Thr161), aurora A and p-PLK1 from Cell Signaling Technology (Beverly, MA, USA); anticyclin B1, CDK1, p-CDK1(Tyr15), and PLK1 from Santa Cruz Biotechnology (CA, USA); anti-BAX from BD Biosciences; anti-p-Ser/Thr-MPM-2 from Millipore; and anti-*α*-tubulin from Sigma-Aldrich. Anti-mouse IgG peroxidase conjugate and anti-rabbit IgG peroxidase conjugate from Sigma-Aldrich were used as secondary antibodies. For immunofluorescence assay, the following primary antibodies were used: anti-AIF from Santa Cruz, anti-*α*-tubulin from Sigma-Aldrich, and antipericentrin from abcam (Cambridge, MA, USA), and anti-mouse-FITC and anti-mouse-TRITC antibody were purchased from Sigma-Aldrich. To stain nucleus, we used DAPI from Sigma-Aldrich.

### 2.3. Cell Viability Assay

Cell lines were treated with 1.0 *μ*M chelidonine for 24 h in 96-well plates and 20 *μ*l/well of CellTiter 96® AQueous One Solution Reagent (MTS; Promega Corporation, WI, USA) was added. After 1.5 h at 37°C in a 5% CO_2_-incubator, absorbance at 490 nm was measured using a Bio-Rad Model 680 microplate reader (Bio-Rad Laboratories).

### 2.4. Flow Cytometry

For cell cycle analysis, cells were harvested and fixed in ice-cold 70% ethanol (Merck, Darmstadt, Germany) overnight at −20°C. The cells were centrifuged to remove the ethanol, washed with phosphate-buffered saline (PBS), suspended in 100 *μ*g/ml RNase (iNtRON Biotechnology, Seongnam, Korea) in PBS, and incubate for 2 h at 37°C, prior to addition of 10 *μ*g/ml propidium iodide (PI; Sigma-Aldrich). For the analysis of mitochondrial potential, cells were stained with 250 nM Mitotracker CMXRos (Invitrogen, CA, USA) for 30min at 37°C, then fluorescence was measured with a FACSVerse™ flow cytometer (BD Bioscience, NJ, USA) and analyzed with FlowJo V10 software (FlowJo, OR, USA).

### 2.5. Cell Cycle Synchronization

Cell cycle synchronization was performed by double thymidine inhibition, as previously described [13]. Briefly, cells were incubated in growth medium to achieve approximately 40% confluency the following day. The medium was replaced with fresh medium containing 2 mM thymidine (Sigma-Aldrich) in PBS for 12 h, and the cells were then three times with PBS and incubated in fresh medium for a further 12 h. After this, the cells were thymidine-treated for a further 12h and then finally incubated in fresh medium containing chelidonine or vehicle.

### 2.6. Western Blot Analysis

Whole-cell lysates were prepared using PRO-PREP™ protein extraction solution (iNtRON Biotechnology, Seongnam, Korea) in the presence of a phosphatase inhibitor cocktail (Set V; Calbiochem, Darmstadt, Germany). The protein content of each cell lysate was quantified using the Bio-Rad Protein Assay system (Bio-Rad Laboratories), then lysates containing equivalent quantities of protein were separated by sodium dodecyl sulfate-polyacrylamide gel electrophoresis (SDS-PAGE) and transferred onto PVDF membranes (EMD Millipore Corporation, MA, USA). Membranes were blocked for 1 h at room temperature with 5% nonfat dry milk (BD Biosciences, NJ, USA) in TBS-T (TBS containing 0.05% Tween 20) and hybridized with the indicated primary antibodies overnight at 4°C. Membranes were then washed three times with TBS-T and incubated with peroxidase-conjugated goat anti-mouse or anti-rabbit secondary antibodies for 1 h at room temperature. Immunoreactive bands were visualized using Clarity™ Western ECL Blotting Substrate (Bio-Rad Laboratories), followed by exposure to X-ray film (CP-BU new; AGFA, Mortsel, Belgium).

### 2.7. Immunofluorescence Assay

For immunofluorescence analysis, T98G cells were grown on a glass coverslip, fixed with 4% paraformaldehyde (BioShop, Burlington, ON, Canada) in PBS, washed with PBS, and permeabilized with 0.1% Triton X-100, before addition of the appropriate primary and secondary antibodies. Microscopy was performed with a confocal laser scanning microscope (FV1000; Olympus, Tokyo, Japan), and the images were captured and processed using FV10-ASW 3.1 Viewer software (Olympus).

## 3. Statistical Analysis

Statistical analysis was performed using the unpaired Student's* t*-test. Results were considered statistically significant if* p *< 0.05. Error bars represent standard deviation derived from at least three independent experiments.

## 4. Results

### 4.1. Chelidonine Induces Apoptosis in the T98G Human Glioblastoma Cell Line

**Figure 1:**
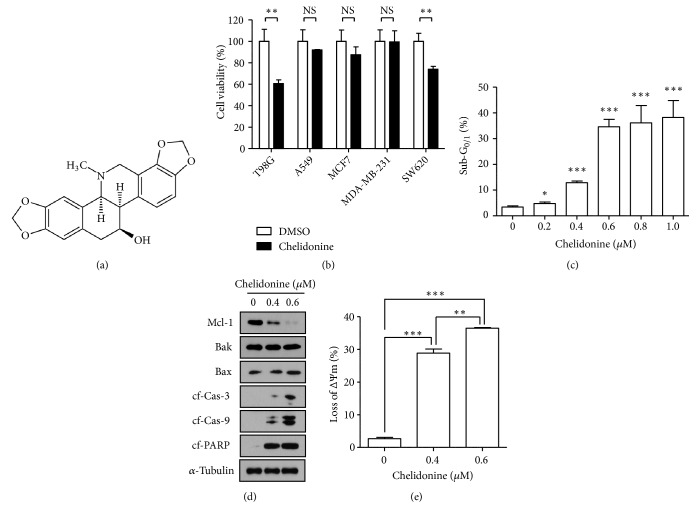
*Chelidonine induces apoptosis in human glioblastoma, T98G cell line.* (a) The chemical structure of chelidonine. (b) Human glioblastoma (T98G), lung cancer (A549), breast cancer (MCF7, MDA-MB-231), colon cancer (SW620) cell lines and noncancer (human embryonic kidney cell: HEK293, human umbilical vein endothelial cell: HUVEC, human fibroblast: CCD-25Sk) were treated with chelidonine (1.0 *μ*M) for 24 h, and a dimethylthiazolyl-carboxymethoxyphenyl-sulfophenyl-tetrazolium (MTS) assay was used to determine cell viability. (c) T98G cells were treated with the indicated concentration of chelidonine for 24 h. The size of the sub G_1/0_ population of T98G cells, indicative of cell death, was determined by PI staining and flow cytometry analysis. (d) Whole T98G cell lysates were subjected to western blot analysis with the indicated antibodies. Cf; cleaved fragment. (e) Mitochondrial depolarization. Cells were stained with MitoTracker Red CMXRos and then analyzed using flow cytometry. Each experimental result represents the mean ± SEM of three independent experiments. *∗∗∗*,* p *< 0.001, *∗∗*,* p *< 0.01, *∗*,* p *< 0.05 by* t*-tests.

To determine whether chelidonine (Figure 1(a)) is cytotoxic in various human tumor cell lines, 1.0 *μ*M chelidonine was added to the indicated cell lines for 24 h, followed by MTS assay. Whereas chelidonine had no or a little effect on survival in most of cells, it reduced the viability of T98G glioblastoma to about 60% (Figure 1(b)). To establish whether chelidonine induces apoptosis in T98G cell line, the sub-G1/0 population of the cells was analyzed on 24 h after treatment with chelidonine. As shown in Figure 1(c), chelidonine induced an increase in the sub-G1/0 population size in concentration-dependent manner. In particular, when 0.6 *μ*M chelidonine was used, the number of sub-G1/0 phase was significantly increased to about 10 times that of the control. To confirm that chelidonine was inducing apoptosis, the expression of apoptosis-related proteins was analyzed by western blotting (Figure 1(d)). In particular, the level of Mcl-1 protein was significantly downregulated, and cleavage of caspase-3 and -9 was induced in chelidonine-treated T98G cells. A reduction of in Mcl-1 protein could lead to a reduction of mitochondrial outer membrane permeabilization (MOMP), which is a cause of apoptosis, and this was demonstrated in chelidonine-treated cells (Figure 1(e)).

### 4.2. Chelidonine Induces Apoptosis via Caspase-Dependent and -Independent Pathways

**Figure 2:**
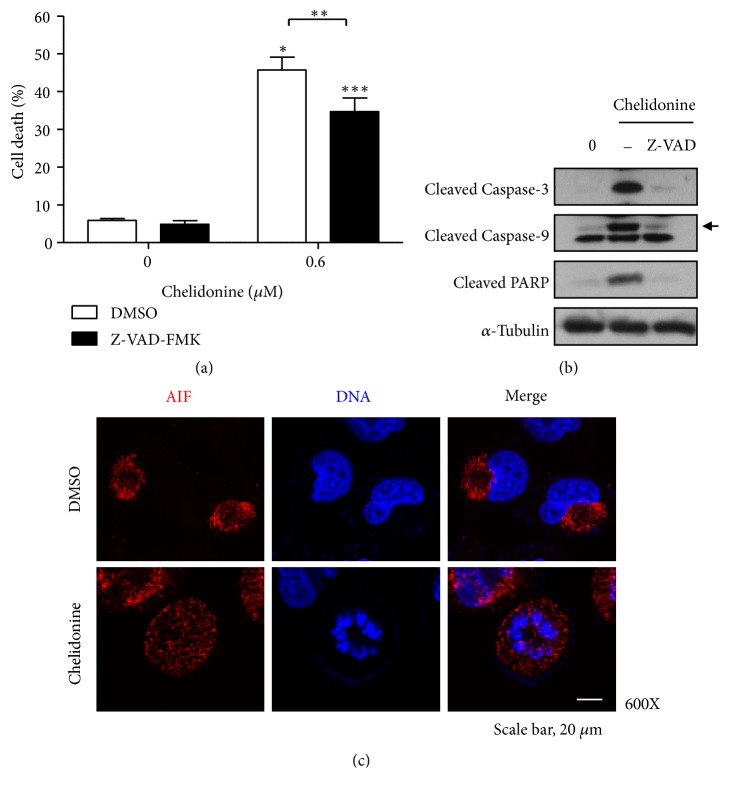
*Chelidonine induces caspase-dependent and -independent apoptosis in T98G cells.* (a) Cells were pretreated with 50 *μ*M Z-VAD-FMK for 1 h, followed by treatment with 0.6 *μ*M chelidonine for 24 h. (b) Whole T98G cell lysates were subjected to western blot analysis with the indicated antibodies. The arrows indicate bands corresponding to cleaved caspase-9. Arrow: cleaved caspase-9. (c) T98G cells were synchronized by double thymidine inhibition, washed, and then incubated with 0.6 *μ*M chelidonine for indicated periods of time after synchronization. They were then immunostained for AIF (red) and DNA (DAPI; blue). Images were captured using confocal laser scanning microscopy. Magnification, 600X. Scale bar, 10 *μ*m.

In order to confirm the induction of caspase-dependent apoptosis by chelidonine, T98G cells were treated with chelidonine in the presence or absence of the pan-caspase inhibitor Z-VAD, and then the size of the sub-G_1/0_ population was determined using PI staining and flow cytometry (Figure 2(a)), and the statuses of caspase-3, caspase-9, and PARP were confirmed (Figure 2(b)). Although the pan-caspase inhibitor could inhibit cleavage of caspase-3, caspase-9, and PARP, apoptosis was only partially inhibited by the inhibitor. These results suggest that chelidonine might induce apoptosis through a caspase-independent pathway, as well as caspase-dependent pathway [14]. To investigate whether caspase-independent apoptosis was induced by chelidonine, the translocation of AIF, a molecular marker, into the nucleus was analyzed after treatment with chelidonine for 24 hours (Figure 2(c)). This showed that AIF did translocate from the cytosol to the nucleus in chelidonine-treated cells, but remained in the cytosol of control cells. Taken together, these findings suggest that chelidonine might induce caspase-dependent apoptosis through caspase-3 and -9, but also caspase-independent apoptosis by inducing AIF translocation into the nucleus.

### 4.3. Chelidonine Induces *G*_2/*M*_ Arrest in the T98G Cell Line

**Figure 3:**
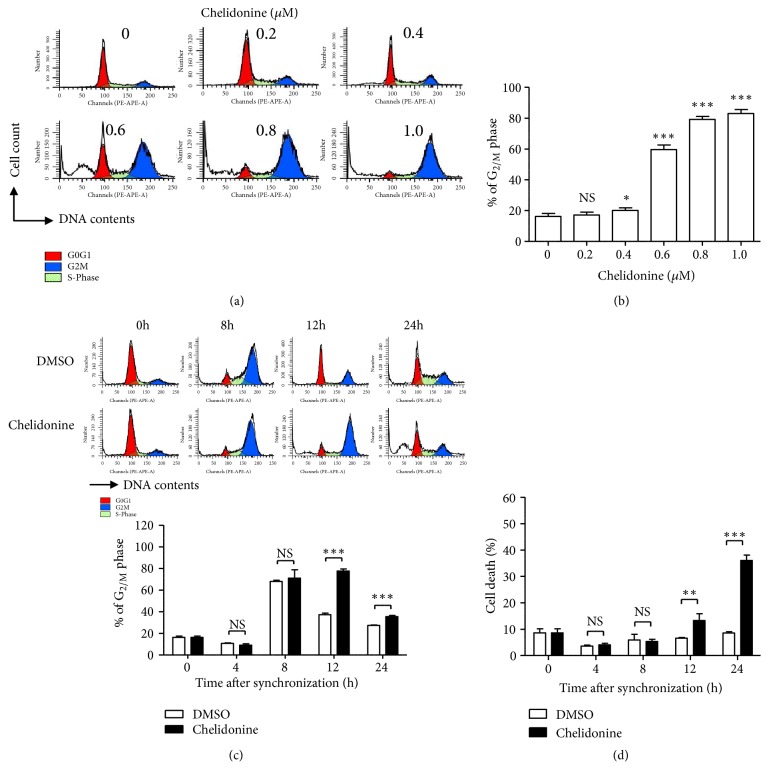
*Chelidonine induces G*_2/*M*_ *arrest in T98G cells.* T98G cells were seeded in six-well plates and incubated with the indicated concentration of chelidonine for 24 h (a). They were then stained with propidium iodide and analyzed with flow cytometry. (b) The numbers of cells in G_2/M_ phase of cell cycle were analyzed using ModFit LT™. (c) T98G cells were treated with 2 mM thymidine for 12 h, the thymidine was removed by washing with PBS (3 times), and fresh media was added to the culture plates for 12 h, after which they were retreated with 2 mM thymidine for 12 h. The G_1/0/_ arrested cells were then released by PBS washing and the addition of fresh medium containing 0.6 *μ*M chelidonine or DMSO for the indicated periods of time. The cell cycle was analyzed at the indicated time points by PI staining and flow cytometry. The data show the percentages of cells in and G_2/M_ phase (c) and sub-G_1/0_ (d). Error bars represent the standard deviation. The data were analyzed using* t*-test. *∗p *< 0.05, *∗∗p *< 0.01, *∗∗∗p *< 0.001.

As a result of the effects described, chelidonine increased the number of cells in G_2/M_ phase in the concentration-dependent manner (Figure 3(a)), in particular when 0.6 *μ*M chelidonine was used (Figure 3(b)). To analyze its effects on the cell cycle more precisely, cells were synchronized at G_1/0_ phase using double thymidine inhibition, before treatment with chelidonine. Vehicle or chelidonine was added after synchronization of the cell cycle, and then the number of cells in each phase of the cell cycle was measured at each indicated time (Figure 3(c)). Control cells were mainly in G_2/M_ phase after 8 h and the cell cycle was completed within about 12 h after synchronization. In contrast, cells treated with chelidonine were mainly in the G_2/M_ phase, not only on 8 h but also on 12 h after synchronization. Twenty-four hours after chelidonine treatment, the number of cells in G_2/M_ phase was lower and the size of the sub-G_1/0_ population had significantly increased (Figures 3(c) and 3(d)). This suggests that apoptosis was induced in cells that do not progress from G_2/M_ phase to G_1/0_ phase.

### 4.4. The Prolonged *G*_2/*M*_ Arrest Induced by Chelidonine Causes Apoptosis in T98G Cells

**Figure 4:**
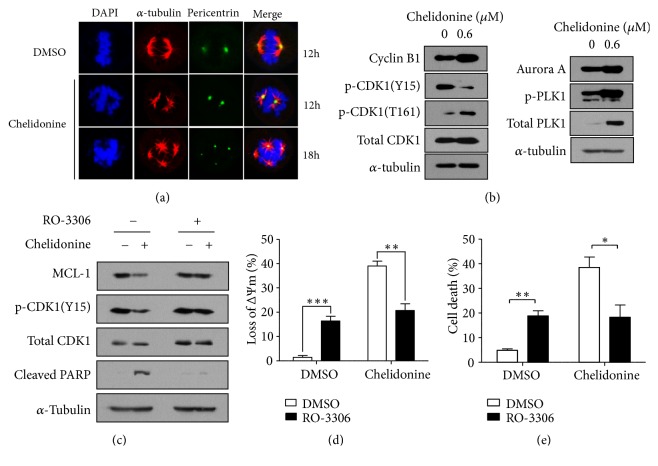
*Chelidonine-mediated G*_2/*M*_ *arrest induces apoptosis in T98G cells.* (a) Chelidonine-mediated multipolar spindle assembly formation. T98G cells were synchronized at G_1/0_ through double thymidine inhibition. After the synchronization, the cells were released and cultured in the presence or absence of 0.6 *μ*M chelidonine for 12 or 18 h. Cells were immunostained for *α*-pericentrin (green), *α*-tubulin (red), and DNA (DAPI; blue). Images were captured using confocal laser scanning microscope. Magnification, 600×. (b) Whole-cell T98G lysates were subjected to western blot analysis with antibodies against cyclin B1, total or phosphorylated CDK1 (Tyr15 and Thr161), aurora A, total and phosphorylated PLK-1 (Thr210). Tubulin served as a loading control. (c) After the synchronization, cells were cultured in the presence or absence of 10 *μ*M RO-3306 and/or 0.6 *μ*M chelidonine for 24 h. And then the whole-cell T98G lysates were subjected to western blot analysis with the indicated antibodies. (d and e) After the synchronization, cells were cultured in the presence or absence of 10 *μ*M RO-3306 and/or 0.6 *μ*M chelidonine for 24 h. And then the mitochondrial potential (d) and the size of sub G_1/0_ population (e) of T98G cells were determined by flow cytometry analysis. Each experimental result is shown as the mean and SEM of three independent experiments. The data was analyzed using* t*-test. *∗p *< 0.05, *∗∗p *< 0.01.

To investigate how chelidonine could induce G_2/M_ arrest in T98G cells, spindle assembly, one of the features of the G_2/M_ phase, was assessed using confocal microscopy (Figure 4(a)). After cell cycle synchronization, the cells were treated with chelidonine for 12 or 18 h, and then spindle assembly and chromosome arrangement were assessed by staining for *α*-tubulin Ab, *α*-pericentrin Ab, and DAPI, respectively. While normal chromosome arrangement and bipolar spindle assembly were observed in vehicle-treated T98Gs, abnormal chromosome arrangement and multipolar spindle assembly were observed in chelidonine-treated cells. This multipolar spindle assembly might be the cause of prolonged G_2/M_ phase and lead some of mitotic cells to cell die. To determine the levels of G_2/M_ marker proteins associated with chelidonine-mediated G_2/M_ arrest, western blotting was performed (Figure 4(b)). This showed that accumulation of cyclin B1, a reduction in inhibitory phosphorylation at Tyr15, and an increase in active phosphorylation at Thr161 of CDK1 were induced by chelidonine. This implies that the cyclin B1/CDK1 complex is activated by chelidonine and therefore that the G_2/M_ phase is prolonged. However, the expressions of aurora kinase A (Aurora A), PLK1, and MPM-2 were also higher in chelidonine-treated cells. In the mitotic phase, active aurora A phosphorylates PLK1, and p-PLK1 activates Cdc25, which then dephosphorylates CDK1 at Tyr15 [15]. Therefore, the activities of Cdc25, CDK1, and cyclin B1/CDK1 complex were maintained longer in the chelidonine-treated cells than in the control cells, providing a mechanism whereby chelidonine might induce an accumulation of T98G cells in G_2/M_ phase.

To establish whether higher activation of CDK1 induced the downregulation of Mcl-1, T98G cells were treated with chelidonine in the presence or absence of RO-3306, CDK1 inhibitor, for 24 h after synchronization. The expression levels of Mcl-1, CDK1, and PARP were then measured by western blot analysis (Figure 4(c)), and MOMP and sub-G_1/0_ population size were determined by PI staining and flow cytometry (Figures 4(d) and 4(e)). In fact, RO-3306 rescued the chelidonine-mediated reduction in Mcl-1 expression and reduced the chelidonine-induced apoptosis and cleavage of PARP. These data suggest that chelidonine-mediated CDK1 is required for apoptosis through Mcl-1 degradation, as well as G_2/M_ arrest.

## 5. Discussion

In this study, chelidonine showed cytotoxicity in T98G glioblastoma cells. Chelidonine induced apoptosis as well as G_2/M_ arrest, which might be responsible for inducing apoptosis. There are three major causes of G_2/M_ arrest, all of which involve the cyclin B1/CDK1 complex [16, 17]. G_2/M_ arrest occurs when (a) the expression of cyclin B1 is reduced and therefore aberrant cyclin B1/CDK1 complexes are formed, (b) phosphorylation and dephosphorylation of CDK1 are not performed normally [18], or (c) the degradation of cyclin B1 does not occur in metaphase [19]. In this study, we present evidence that the accumulation of cyclin B1 and abnormal phosphorylation/dephosphorylation of CDK1 induce prolongation of G_2/M_ (Figure 4(b)). Cyclin B1 is degraded by the E3 ubiquitin ligase, APC/C, in metaphase after activation of the cyclin B1/CDK1 complex [20]. Therefore, we propose that chelidonine induces G_2/M_ arrest by interfering metaphase in T98G cells.

Interphase, prophase, prometaphase, metaphase, anaphase, telophase, and cytokinesis take place in that order during the G_2/M_ phase. Immunofluorescence analysis showed multipolar spindle assembly in cells treated with chelidonine (Figure 4(a)). This event occurs when the spindle assembly checkpoint (SAC) of the G_2/M_ phase is performed abnormally [21]. The SAC is turned on during prometaphase and normally turned off after metaphase is completed. At this stage, various proteins, including cyclin B1, are ubiquitinated by activated APC/C, which allows the cells to proceed with metaphase to anaphase transition [22]. In this study, it is likely that multipolar spindle assembly was the result of chelidonine-induced inactivation of APC/C, causing the accumulation of cyclin B1 and G_2/M_ arrest. This multipolar spindle assembly influences a variety of events, mainly inducing apoptosis by mitotic slippage, mitotic catastrophe, or rarely by causing abnormal cell division [23].

For apoptosis to occur, the antiapoptotic protein, Mcl-1, is degraded, and the proapoptotic proteins, BAK and BAX, are activated, resulting in mitochondrial outer membrane permeabilization (MOMP). Damaged mitochondria induce apoptosis by releasing cytochrome C, endonuclease G, and AIF into the cytosol. Cytochrome C interacts with Apaf-1 to form apoptosome with procaspase-9, which activates caspase-3 and induces apoptosis through PARP cleavage (caspase-dependent apoptosis) [24]. However, endonuclease G and AIF can also translocate to the nucleus and cause DNA fragmentation, leading to caspase-independent apoptosis [25]. In this study, it was shown that cleavage of caspase-3, caspase-9, and PARP and translocation of AIF from mitochondria (cytosol) to the nucleus occur when T98G cells were treated with chelidonine. This suggests that chelidonine induces both caspase-dependent and -independent apoptosis. Mcl-1, which is located on the mitochondrial outer membrane, binds BAK and BAX and then inhibits their activities [26]. Mcl-1 is phosphorylated by various kinases such as GSK3*β*, JNK, CDK1, and p38 [27] and then degraded by the ubiquitin proteasome system. In particular, phosphorylation of Mcl-1 by CDK1 might be a key bridge between G_2/M_ arrest and apoptosis [28].

Taking these results together, chelidonine induces G_2/M_ arrest through multipolar spindle assembly causing G_2/M_ arrest in T98G cells, which likely induces both caspase-dependent and -independent apoptosis through Mcl-1 degradation. These findings suggest that chelidonine might represent a lead compound for anticancer chemotherapy.

## Data Availability

The data used to support the findings of this study are available from the corresponding author upon request.
